# Investigation on 3D Printing of Shrimp Surimi Adding Three Edible Oils

**DOI:** 10.3390/foods13030429

**Published:** 2024-01-29

**Authors:** Yanmo Pan, Qinxiu Sun, Yang Liu, Shuai Wei, Zongyuan Han, Ouyang Zheng, Hongwu Ji, Bin Zhang, Shucheng Liu

**Affiliations:** 1 College of Food Science and Technology, Guangdong Ocean University, Guangdong Provincial Key Laboratory of Aquatic Products Processing and Safety, Guangdong Province Engineering Laboratory for Marine Biological Products, Guangdong Provincial Engineering Technology Research Center of Seafood, Guangdong Provincial Engineering Technology Research Center of Prefabricated Seafood Processing and Quality Control, Zhanjiang 524088, China; 2 Collaborative Innovation Center of Seafood Deep Processing, Dalian Polytechnic University, Dalian 116034, China; 3 College of Food Science and Pharmacy, Zhejiang Ocean University, Zhoushan 316022, China

**Keywords:** shrimp surimi, oil, three dimensional printing, physical properties, rheological properties, textural properties

## Abstract

Three-dimensional (3D) printing provides a new method for innovative processing of shrimp surimi. However, there still exists a problem of uneven discharge during the 3D printing of surimi. The effects of different amounts of lard oil (LO), soybean oil (SO), and olive oil (OO) (0%, 2%, 4%, and 6%, respectively) added to shrimp surimi on the 3D printability of surimi were evaluated. The findings showed that with the increase in the added oil, the rheological properties, texture properties, water-holding capacity (WHC), and water distribution of surimi with the same kind of oil were significantly improved; the printing accuracy first increased and then decreased; and the printing stability showed an increasing trend (*p* < 0.05). The surimi with 4% oil had the highest printing adaptability (accuracy and stability). Different kinds of oil have different degrees of impact on the physical properties of surimi, thereby improving 3D-printing adaptability. Among all kinds of oil, LO had the best printing adaptability. In addition, according to various indicators and principal component analysis, adding 4% LO to shrimp surimi gave the best 3D-printing adaptability. But from the aspects of 3D printing properties and nutrition, adding 4% SO was more in line with the nutritional needs of contemporary people.

## 1. Introduction

Three-dimensional (3D) printing enables the shaping of personalized shapes that are difficult to make with conventional mills [1,2]. At present, it is widely used in food processing, such as chocolate [3], mashed potato [4], potato starch [5], soy protein isolate [6], and so on. Aquatic products are rich in protein, polyunsaturated fatty acids, and trace elements, with less fat and low calories [7,8]. Currently, other printing properties based on seafood-based ink bases and the properties of the developed 3D structures are continuously being investigated [9,10,11].

*Litopenaeus vannamei* has a short reproductive cycle, fast growth rate, and wide environmental adaptability, so it has high commercial value [12]. The surimi is a high-value product of shrimp. At present, shrimp surimi products are popular among modern consumers, but surimi products are relatively simple in shape, which cannot meet the needs of consumers for diversified appearance. Shrimp surimi has a relatively dense gel structure, and it has the characteristics of raw materials suitable for 3D printing [11]. However, previous studies [13] have found that pure shrimp surimi was prone to uneven discharge and easily undergoing sedimentary collapse in the 3D-printing process. This is mainly due to the poor fluidity and support of the surimi. Lipids can interact with proteins during the formation of a meat gel system and occupy space in the protein gel matrix network, thus increasing the printing support performance of surimi. In the study of Xie et al. [14], the addition of linseed oil to the printed cod protein compound gel can enhance the continuity and chewability of the extrusion material. Zhou et al. [15] reported that camellia oil reduces porosity in gelled meat products and alters the protein network structure, resulting in a stronger structure to increase the support of the gel. Adding oil can improve the material properties of samples with poor fluidity and support, and make the material flow out of the nozzle smoothly, thus increasing printing accuracy [16,17,18]. In addition, oils and fats are also commonly added to shrimp surimi products to improve the flavor and texture properties of products [8,19]. Therefore, fats and oils can be added to shrimp surimi to improve its material properties to make it suitable for 3D printing. Different kinds and contents of lipids will greatly affect the rheological and textural properties of materials, and further affect the 3D printability of materials [20].

As one of the most important animal fats, lard oil (LO) has a very good stability and can be better combined with gel-based products in theory. However, at present, high cholesterol, hypertension, and hyperlipidemia are becoming hotspots of attention. Vegetable oils are more in line with the requirements of the population for healthy eating. Lard oil (LO), soybean oil (SO), and olive oil (OO) are three typical oils. Therefore, shrimp surimi with different kinds (LO, SO, and OO) and contents (0%, 2%, 4%, and 6%, respectively) of oils added was prepared in the present study. The 3D printability, physical properties, correlation and principal component analysis (PCA) of surimi were evaluated in the present study. In this study, the kinds of oils and the amount of added oils were determined to improve the adaptability of 3D printing surimi, to meet the needs of consumers’ personalized nutritional diet, to improve the added value of surimi products, and to provide a theoretical basis for the application of 3D printing surimi.

## 2. Materials and Methods

### 2.1. Materials

Salt (Guangdong Salt Industry Group Co., Ltd., Guangzhou, China), lard oil (Gaojin Food Co., Ltd., Suining, China), soybean oil (Yihai Kerry Arawana Holdings Co., Ltd., Shanghai, China), and olive oil (Spain Oriel Import and Export Trading Co., Ltd., Sevilla, Andalusia, Spain) were purchased from Lotus Supermarket (Zhanjiang, China).

### 2.2. Preparation of Surimi

*Litopenaeus vannamei* (30–40 individuals/kg) were purchased from the Huanle Ocean Aquatic Market (Zhanjiang, China). The shrimps were then stunned in an ice bath, and after removing the heads, shells, and intestinal glands, they were placed in a blender (GZB20, Shanbao Food Factory Machinery Department, Guangzhou, China) and blended for 5 min, and added with 2% salt to grind for 2 min. After that, the samples were divided into ten equal parts. Nine of them were added with LO (2%, 4%, and 6%), SO (2%, 4%, and 6%), and OO (2%, 4%, and 6%), respectively, then, these samples were ground for 2 min again. And the other part of the original sample without any oil added was set as the control group.

### 2.3. The 3D Printability

The assessment of 3D printability follows the method described by Pan et al. [13]. The 3D printing model was designed for a cube. The side length was 20 mm. We set the printing parameters of the 3D printer (FOODBOT E1, Shinnove, Hangzhou, Zhejiang, China): the temperature of the barrel was 25 °C, the nozzle diameter was 1.2 mm, the printing speed was 30 mm/s, the printing height was 2 mm, and the filling rate was 80%. After printing, the plane side length (*L*_s_, mm) and height (*H*_0_, mm) of the 3D-printed products were measured by vernier calipers. At the same time, the 3D-printing products were evaluated in terms of their appearance. The printing accuracy (%) was calculated through Equation (1). The printed product was placed for 60 min, and the height of the printed product was measured (*H*_60_, mm). The printing stability (%) was calculated through Equation (2).

(1) $$\mathrm{The}\;\mathrm{printing}\;\mathrm{accuracy}\;(\%)=(1-\left|\frac{L_{s}-L_{m}}{L_{m}}\right|)\times 100$$

(2) $$\mathrm{The}\;\mathrm{printing}\;\mathrm{stability}\;(\%)=\frac{H_{60}}{H_{0}}\times 100$$

where *L*_s_ was the side length of the products, mm; *L*_m_ was the side length of the model, mm; *H*_0_ was the height of the products placed for 0 min, mm; *H*_60_ was the height of the products when they were placed for 60 min, mm.

### 2.4. Rheological Properties

We referred to the method of Liu et al. [10] to test the rheological properties of surimi. The HAAKE MARS Ⅲ modular advanced rheometer (Thermo Fisher Scientific, Waltham, MA, USA) was used to coat the sample on a circular specimen bench with a diameter of 35 mm. The samples were coated on a circular sample table with a diameter of 35 mm. A P35Ti L rotor was used with a gap of 1 mm between it and the sample table. The rheometer was at 25 °C in temperature and the shear rate ranged from 0.1 to 100 s^−1^.

Dynamic moduli measurement was achieved by the small amplitude oscillation frequency scanning mode. The frequency was set to 0.1~10 Hz to achieve the storage modulus (*G*′, Pa) and loss modulus (*G*″, Pa) of the samples, and the oscillation frequencies were recorded. All measurements were carried out in a certain viscoelastic linear region. The strain scanning was 0.1%.

### 2.5. Textural Properties

TMS-Pro texture tester (Food Technology Corporation, Sterling, VA, USA) was used to test the texture properties of surimi, referring to the method of Pan et al. [13]. Texture profile analysis was conducted on the hardness, springiness, and adhesiveness of surimi under the conditions of 25 °C and 40~50% humidity.

### 2.6. Water Distribution

A low-field nuclear magnetic resonance (LF-NMR) (NMI20-060H-I, Niumag Electric Corporation, Shanghai, China) was used to measure the transverse relaxation time (*T*_2_) of surimi. The resonance frequency and magnetic field intensity of the instrument were set to 22.6 MHz and 0.47 A/m, respectively. The transverse relaxation time *T*_2_ was obtained, the number of iterations was 10^5^, and the obtained curve inversion was *T*_2_.

### 2.7. Water-Holding Capacity

Referring to the method of Liu et al. [21], a certain mass of surimi (*W*_1_, g) was weighed, centrifuged at 4 °C (10,000× *g*) for 10 min, and then filter paper was used to absorb the water on the surimi surface and weighed (*W*_2_, g). The calculation of WHC was as shown in the following Equation (3):

(3) $$\mathrm{WHC}\;(\%)=\frac{W_{2}}{W_{1}}\times 100$$

### 2.8. Statistical Analysis

Experimental data are presented as the mean ± standard deviation. Two-way ANOVA and Tukey’s multiple comparison test were used to determine the significance between the means at a confidence level of 95%. A total of three batches of surimi were produced. The texture test was repeated 5 times for each batch and the other tests were repeated 3 times.

## 3. Result and Discussion

### 3.1. The 3D Printability

**Figure 1:**
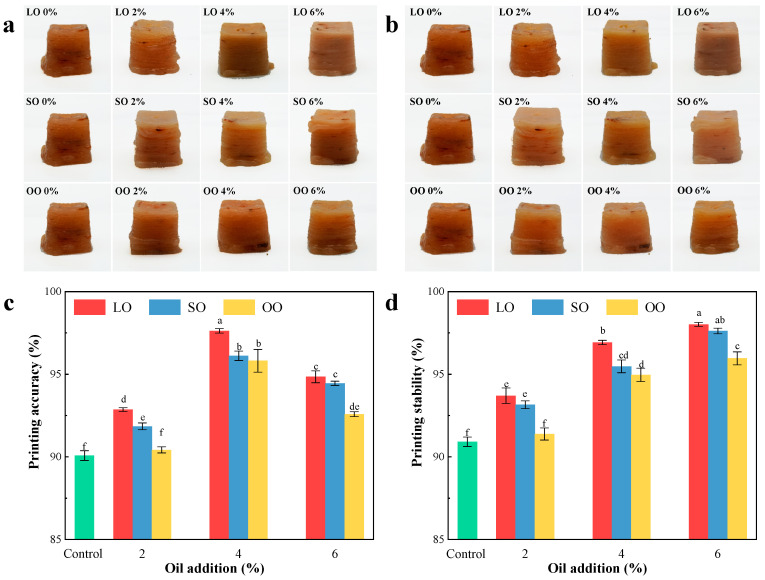
Effects of adding different kinds of oil to surimi on 3D-printing adaptability. (**a**,**b**) 3D-printed surimi with different kinds of oil added at 0 min and 60 min, photos; (**c**,**d**) Effects of different kinds of adding oil on the printing accuracy and stability of the 3D surimi structure. (LO, lard oil; SO, soybean oil; OO, olive oil, different letters in the same indicator indicate significant differences (*p* < 0.05).

The printing adaptability (accuracy and stability) represents important indicators to evaluate the quality of 3D-printing products [10]. By comparing Figure 1a,b, it can be seen that different degrees of collapse occurred after the product was placed for 60 min. The 3D-printing appearance of the control (without oil) was the worst due to the appearance of broken filaments and sedimentation collapse during the printing process. Adding 2% oil could improve the printing accuracy of the surimi, but there were still some problems such as the uneven outflow of material, stiff printing lines, and poor blending between layers. Adding an appropriate content of oil (4%) effectively improved the 3D-printing structure of the surimi, making the surfaces smooth, with a high printing accuracy, fine lines of the printing products, and without obvious deposition collapse and other phenomena. This may be due to the strong gel binding ability of oil, which can improve the emulsifying properties of the surimi [22,23]. However, when adding too much oil (6%), the printing effects of the samples were poor. This may be due to the excessive addition of oil; the sample fluidity was better, resulting in the discharge speed of the surimi being too fast, and the sudden outflow of the samples, so that the printing accuracy decreased. Figure 1c,d summarized the printing adaptability. In accordance with Figure 1c, the 3D-printing accuracy of surimi with the same kind of oil increased and then decreased as the oil content increased (*p* < 0.05). In addition, as shown in Figure 1d, the printing stability of the samples showed a gradually increasing trend as the amount of oil added increased (*p* < 0.05). This may be due to the change in protein structure caused by the increase in oil content, and the void inside the protein network being filled with oil, thus limiting the movement of the protein matrix in the network and forming a denser network structure [24], so the WHC and hardness of surimi will gradually increase, and the corresponding support and stability will gradually increase as well. For the samples with the same content of oil, the LO sample had the highest printing adaptability, followed by the SO and OO samples (*p* < 0.05). This may be explained by the differences in fatty acids for different kinds of oil. LO is an animal oil rich in saturated fatty acids, cellular water can be formed on the surface, and its structure is relatively uniform and dense, so that the ability to maintain water is stronger and can build widely near hydrophilic surfaces such as the surface of surimi [25]. Therefore, it has a better WHC than SO and OO, which are rich in unsaturated fatty acids, thereby improving 3D-printing adaptability. In short, compared with other samples, shrimp surimi with LO has better support and stability, and adding 4% LO can effectively improve the 3D-printing adaptability of surimi.

### 3.2. Rheological Properties

**Figure 2:**
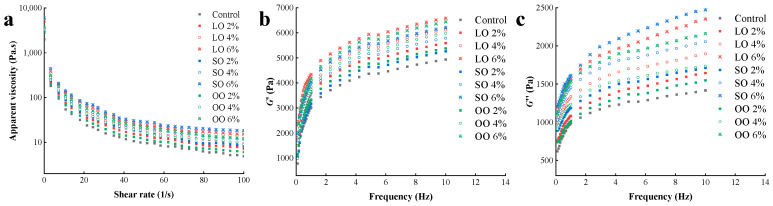
Changes in (**a**) apparent viscosity, (**b**) storage modulus (*G*′), and (**c**) loss modulus (*G*″) of surimi after adding different kinds of oil.

In extrudation-based 3D printing, shrimp surimi is subjected to relatively high pressure and shear forces through the nozzle. Therefore, the rheological properties of surimi play a crucial role in 3D printing and significantly affect the 3D-printing adaptability [26]. As can be seen in Figure 2a, the apparent viscosity of shrimp decreases with the increase in shear rate, and the apparent viscosity is higher when the shear rate is lower, indicating that all surimi of shrimp were pseudo-plastic fluids with shear thinning [27]. With the increase in oil content, the apparent viscosity increases gradually. This may be because the increase in oil content causes more oil to be emulsified, and the protein matrix expands in the emulsion, resulting in increased viscosity [28]. On the other hand, oil emulsifies surimi particles to form aggregate and network structures that help to resist shear and lead to the higher apparent viscosity observed [29]. Shi et al. [30] studied the effects of different oils on the physical properties of surimi and found that adding oil increased the texture and rheological properties of surimi products. Among samples with the same amount of oil, the SO group had the highest apparent viscosity, which may be attributed to differences in oil phase viscosity [31]. Therefore, the surimi had a very high viscosity. Combined with the effect and appearance image of products (Figure 1a,b), appropriate apparent viscosity was conductive to smoothly extrude 3D-printing materials of surimi from the ink cartridge and increased the fusion between layers. Adding 4% LO was the most instructive to the smooth extrusion of surimi from the ink cartridge and improving the adaptability of the 3D printing of surimi.

In the linear viscoelastic region (Figure 2b,c), the *G*′ of all the surimi was much higher than *G*″, indicating that shrimp surimi had a good springiness and behaved like a solid with poor fluidity. Thus, it had the potential to form an elastic gel or gelatinous structure [11]. It can be seen from Figure 2b,c that *G*′ and *G*″ have similar changes in all samples. As the oscillation frequency increases, both *G*′ and *G*″ gradually increase. Furthermore, *G*′ and *G*″ gradually increased with the increase in oil quantity at any oscillation frequency. This may be because the high oil content of the surimi system increased the area of the interfacial film, stabilized the interfacial film through hydrophobic interactions and disulfide bonds, and improved the intermolecular crosslinking of the bulk phase, thereby increasing its *G*′ and *G*″ [32]. Pietrowski et al. [23] found that adding oil rich in ω-3 polyunsaturated fatty acids improved the rheological and textural properties of Alaska pollock surimi, which is consistent with our research. The *G*′ in the LO group was the highest, and the SO group had the highest *G*″. This may be due to fatty acids with different saturation degree causing changes in the spatial conformation and intermolecular interactions of the proteins, thus resulting in differences in the *G*′ and *G*″ of the surimi system [33]. Adding oil increases the *G*′ and *G*″ of the samples, and proper *G*′ and *G*″ will facilitate the flow of material through the cartridge and increase the fusion between layers of printed material [34], thus improving the adaptability of 3D printing.

### 3.3. Textural Properties

**Figure 3:**
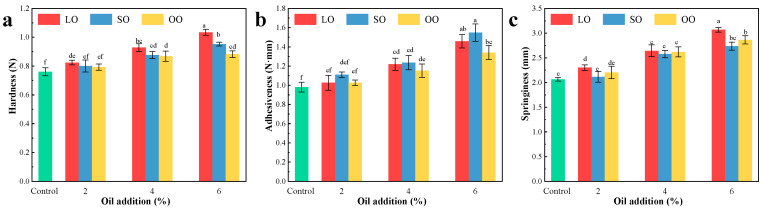
Effects of different kinds of added oil on (**a**) hardness, (**b**) adhesiveness, and (**c**) springiness of surimi. (LO, lard oil; SO, soybean oil; OO, olive oil, different letters in the same indicator indicate significant difference (*p* < 0.05).

The texture characteristics of the surimi are shown in Figure 3. The hardness of all samples increased with increasing oil content (*p* < 0.05) (Figure 3a). The hardness of the control sample was 0.76N, and the hardness values of the surimi added with 6% LO, SO, and OO were 1.03, 0.95, and 0.88N, respectively. This may be because the oil can form smaller particles to fill in the swollen fibers of the surimi, forming a more stable system during the blending process, reducing the precipitation of water, thus increasing the hardness of the surimi [35]. Debusca et al. [36] found that the texture properties of surimi after adding linseed oil, herring oil, and algae oil were improved compared with the control group. The hardness of the LO group was the highest, followed by SO and OO (*p* < 0.05). Combined with the printability, high hardness was conducive to maintaining the original shape of the printed products, thereby improving the printing stability of products. Although samples containing 6% oil have the highest hardness, the sample will block the ink cartridge, resulting in uneven material flow and sudden extrusion of a large amount of material.

With the increase in oil content, the adhesiveness of the samples increased gradually (*p* < 0.05) (Figure 3b). Yang et al. [37] found that the combination ability of water and oil can be improved by increasing the content of oil added, thus increasing the viscosity of the system. The adhesiveness of the SO group was significantly higher than that of other groups (*p* < 0.05). The high viscosity of surimi with added oil can be attributed to the thickening property of the oil dispersed in the surimi system as a thickening agent [16]. The adhesiveness of the 6% SO group was 1.55 N·mm, which was significantly higher than that of the control group (0.98 N·mm) (*p* < 0.05). Thus, the adhesiveness of the surimi was enhanced. Combined with 3D-printing properties, appropriate adhesiveness was beneficial to the fusion of the printed sample layer by layer.

With the increase in oil content, the springiness of the samples increased gradually (*p* < 0.05) (Figure 3c). The springiness of the 6% LO, SO, and OO groups were 3.07, 2.74, and 2.86 mm, respectively, which were significantly higher than that of control group (2.06 mm) (*p* < 0.05). The increase in the springiness was partly because of oils, which filled the gap of the protein network of the surimi, limiting the movement of the protein matrix in its network structure, with the formation of the gel network structure being more compact and more stable, thus increasing the springiness of the surimi. In addition, as the oil content increases, protein will be inserted into the oil droplets, and more protein will be exposed to the hydrophobic side chain and participate in the formation of network structure, thus changing the structure of surimi protein and resulting in stronger lipoprotein interaction, which will also increase the springiness of the surimi [15]. Álvarez et al. [38] found that adding vegetable oil could improve the springiness of protein gels by enhancing their network structure. Wu et al. [39] added oil to the myofibril system and found that its internal network structure was more dense and had a higher springiness.

Different kinds and content of oils can directly affect texture properties, especially the texture of a gel system of surimi and oil. In consequence, it was important to choose appropriate oil to ensure the surimi has an appropriate hardness, adhesiveness, and springiness, so that shrimp surimi can be extruded and discharged smoothly, thus improving the printing features of surimi.

### 3.4. Water Distribution

**Figure 4:**
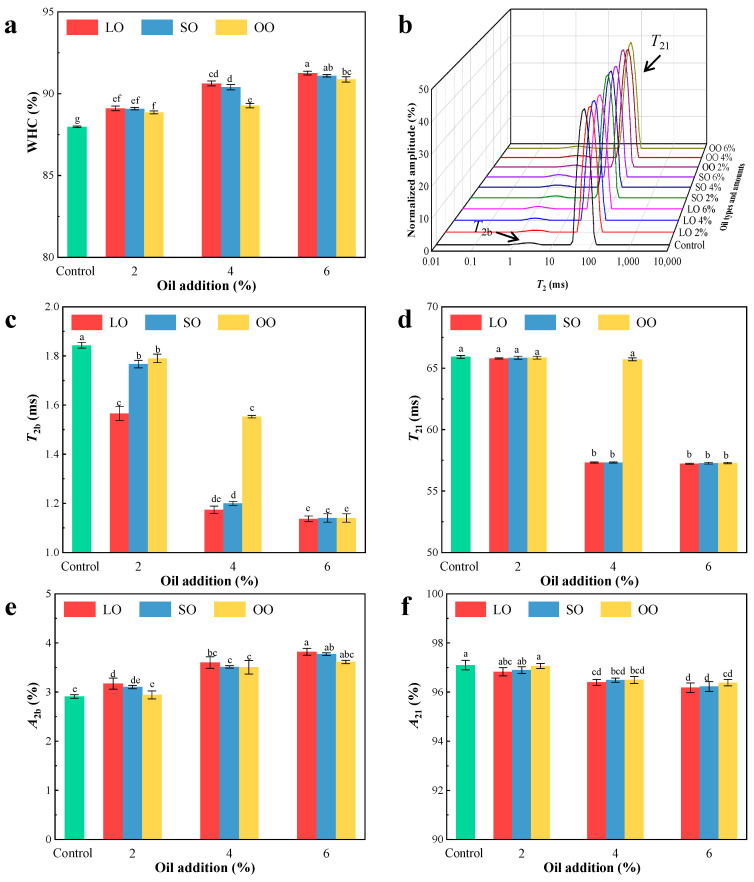
(**a**) Effects of different kinds of added oil on WHC of surimi; (**b**–**f**) Effects of different kinds of oil on the water distribution of surimi. (LO, lard oil; SO, soybean oil; OO, olive oil). Different letters in the same indicator indicate significant differences (*p* < 0.05).

The distribution of moisture plays an important role in the support performance of the printed products and the smoothness of the extrusion process [40]. As can be visualized from Figure 4b, there were two peaks in the LF-NMR curves of surimi, indicating that shrimp surimi protein limited water mobility in different amplitudes, centered at approximately 0~10 ms (*T*_2b_) and 10~100 ms (*T*_21_). *T*_2b_ represents bound water, tightly combining with gel network structure; *T*_21_ represents partially immobilized water, where the binding ability for the gel network structure is weak. Figure 4c–f show the effect of oil on the water relaxation time (*T*_2_) and peak area ratio of amplitude (*A*_2_) for the two kinds. As can be seen from Figure 4, for the samples with the same kind of oil added, the *T*_2_ relaxation time of the samples moved to the left with the increase in oil added, and the peak area ratio of amplitude of partially immobilized water (*A*_21_) gradually decreased, while the peak area ratio of amplitude of bound water (*A*_2b_) gradually increased. It indicated that the addition of oil significantly increases the binding ability of protein and other components, restricts the movement of protons, and makes oil and water bind more closely [41]. This may be because the hydrophobic interaction between the added oil and the protein destroyed the original network space structure, thereby improving the ability of surimi to bind to water [42]. With the increase in content added, the oil can be used as the filler of the surimi network, resulting in a closer microstructure with fewer voids [43], which enhances the ability of surimi to bind water. Among the samples with the same amount of oil added, the LO group had the highest *A*_2b_. This result was related to the WHC and hardness of the surimi, indicating that the addition of oil mainly improves the WHC of surimi by improving the binding force of surimi.

### 3.5. Water-Holding Capacity

WHC is an important indicator to evaluate the quality of modified surimi products. WHC can be used to evaluate the degree of water binding of myofibril in shrimp surimi [21]. It can be observed from Figure 4a that the WHC of the samples added with the same oil increased with the increase in oil content (*p* < 0.05). This suggests that the addition of oil led to a reduction in the water mobility in the surimi [1,14]; the tails of heavy meromyosin faced the aqueous phase, thus forming an interfacial protein film around fat globules, which stabilized the water so as to increase the WHC of surimi [44]. In addition, Zhou et al. [20] found that the addition of lipids significantly increased the WHC of surimi myofibrillar gels due to the fact that during homogenization, lipids in a certain concentration range were chopped into spheres and then used as fillers in the matrix network of the protein gels. The WHC of the LO group was higher than that of other groups (*p* < 0.05); this may be due to the fact that lard (solid at room temperature) is a conventional plastic fat with different physical properties than soybean oil and olive oil, resulting in a stronger WHC. The result of WHC for shrimp surimi with added oil was similarity with the change of hardness and water distribution. Özogul et al. [45] also discovered by adding vegetable oil to sea bass that the WHC of samples added with SO rich in polyunsaturated fatty acids was higher than that of adding OO rich in monounsaturated fatty acids, which may be due to the stronger thermodynamic stability of SO and water, and the stable interface membrane of surfactant molecules. Therefore, the WHC of SO was higher than OO. Combined with 3D printability, the addition of oil improved the WHC of surimi, provided better support for printed products, and improved printing adaptability.

### 3.6. The Correlation and Principal Component Analysis

**Figure 5:**
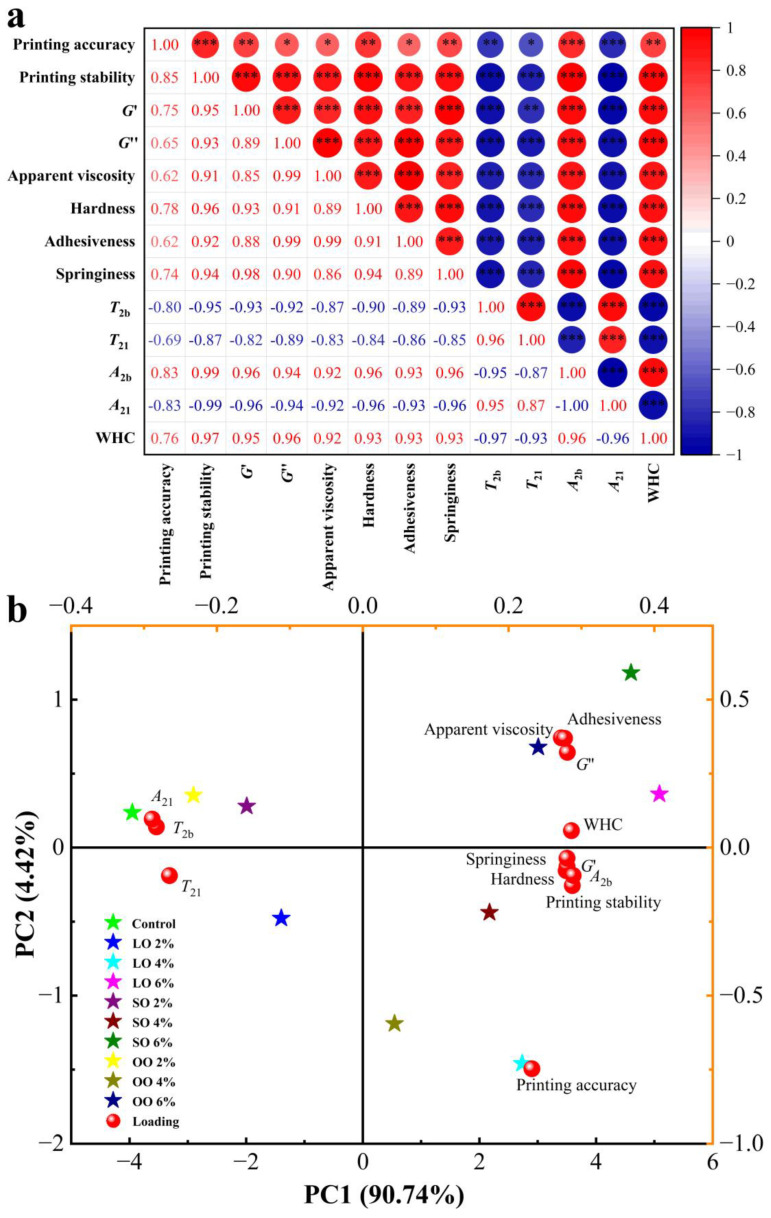
(**a**) Correlation of various index variables with different kinds of oil added, and (**b**) principal component analysis of shrimp surimi with different oil addition kinds. (* means *p* < 0.05, ** means *p* < 0.01, *** means *p* < 0.001, significant effect; LO, lard oil; SO, soybean oil; OO, olive oil).

Correlation analysis is the analysis of two or more elements of a variable that are correlated to measure the degree of correlation between two elements of the variable. The indicators of surimi such as printing adaptability were assessed. As can be seen from Figure 5a, where red represents a positive correlation and blue represents a negative correlation, these variables all present a good correlation and are significant (*p* < 0.05). This shows that the 3D-printing adaptability of a material is not determined by the rheological, physical, and chemical properties of individual components but determined by the interaction of all components [46].

PCA was used to establish the relationship between different kinds of oils and the content of oils added, 3D-printing adaptability, and the material properties of surimi. As shown in Figure 5b, the contribution rate of Principal Component 1 (PC1) is 90.74%, and that of Principal Component 2 (PC2) is 4.42%, with a cumulative contribution rate of 95.16%. The principal component contribution rate was greater than 85%, indicating that the information expressed in the original data was fully interpreted. Figure 5b shows the loading diagram of PCA, and the feature vectors of PC1 were printing adaptability, rheological properties, textural properties, and water properties. The eigenvectors of surimi were distributed in Quadrants 1, 2, 3, and 4. The printability of the sample was negatively correlated with *A*_21_, *T*_2b_, and *T*_21_ on both sides of Principal Component Axis 1. The other indexes and the printability of the sample were positively correlated with the same side of Principal Component 1.

On the other hand, Figure 5b shows the score plots for the different treatment groups projected in two-dimensional space using PC1 and PC2 as loading factors. The samples located in Quadrants 1, 2, 3, and 4 were inferred to be associated with the variables in the corresponding quadrants [13]. By comparing the score diagram with the load diagram, the printing adaptability of the control group without oil and the group with 2% oil were located on both sides of the coordinate axis of Principal Component 1 and far away from each other. This also proved that the printing adaptability of surimi with no oil or a small content (2%) of oil was relatively poor. The groups with samples added with 4% and 6% oil were in the positive direction of Principal Component 1, indicating that these samples had a higher printing adaptability. In addition, all the samples containing 4% oil were in the same quadrant (Quadrant 4), and printing adaptability was also in the same quadrant, indicating that adding an appropriate amount of oil (4%) to the surimi can improve the printing adaptability of the product. The printing adaptability of the 4% LO group was the closest to all groups, which further proved that adding 4% LO effectively improved the printing adaptability of surimi.

## 4. Conclusions

Adding oil can not only improve the physical properties and other material properties of surimi, but also facilitate the smooth extrusion of materials from the nozzle, thus increasing the 3D-printing adaptability (accuracy and stability) of surimi. The printing adaptability of adding 4% oil was significantly higher than those of other contents. Different kinds of oil and the content of oil added had an impact on the 3D-printing effect. LO had the best printing adaptability among all kinds of oil. However, there was a great restriction for the diet of the population. Compared with various indexes of LO, SO also had good values. Therefore, considering the nutritional properties of these three oils, adding 4% SO to shrimp surimi can properly improve its 3D-printing adaptability.

## Data Availability

The original contributions presented in the study are included in the article, further inquiries can be directed to the corresponding author.
